# Olmesartan-associated gastroduodenitis that was detected on endoscopic follow-up

**DOI:** 10.1007/s12328-025-02137-8

**Published:** 2025-05-04

**Authors:** Yusuke Oki, Takayoshi Yamada, Mai Tatsuno, Mitsuko Iguchi, Hideyuki Miyachi, Kazushige Uchida

**Affiliations:** 1 Department of Gastroenterology and Hepatology, Kochi Medical School, Kochi University, Kohasu, Oko-Cho, Nankoku, Kochi 783-8505 Japan; 2 Department of Gastroenterology, Kuniyoshi Hospital, Kochi, Japan; 3 Department of Internal Medicine, Ooida Hospital, Sukumo, Japan; 4 Department of Diagnostic Pathology, Kochi Medical School, Kochi University, Nankoku, Japan

**Keywords:** Olmesartan, Gastritis, Duodenitis

## Abstract

We present a rare case of olmesartan-associated gastroduodenitis in a 65-year-old female with predominant upper gastrointestinal symptoms and significant changes in endoscopic findings over a short period. The patient presented with epigastralgia and nausea but no diarrhea. Esophagogastroduodenoscopy (EGD) performed 26 months earlier showed a duodenal ulcer and partial mucosal atrophy in the lower gastric body. EGD conducted at our hospital revealed diffuse atrophic mucosa throughout the stomach and fragile mucosa from the middle gastric body to the antrum, with white purulent adhesions. Diffuse inflammatory changes were also observed in the duodenal bulb and descending part. Mucosal biopsy showed atrophic changes, inflammatory cell infiltrations, and epithelial detachments in both the stomach and duodenum. Colonoscopy findings were normal. After discontinuing olmesartan, the patient’s symptoms improved immediately. A follow-up EGD conducted 4 months after discontinuation revealed marked improvement in the stomach and duodenum. At 16 months post-discontinuation, the patient remained asymptomatic, with minimal inflammation and residual gastric atrophy observed on EGD. This case underscores the importance of considering olmesartan as a potential cause of gastroduodenitis, particularly when symptoms and endoscopic findings improve after discontinuation of the medication.

**Supplementary Information:**

The online version contains supplementary material available at 10.1007/s12328-025-02137-8.

## Introduction

In recent years, some studies have reported the development of enteropathy caused by angiotensin II receptor blocker (ARB), particularly olmesartan [1–3]. Sprue-like enteritis attributed to olmesartan was first reported in 2012 [4]. The incidence of olmesartan-associated enteropathy (OAE) is 1 in 2232 patients (< 0.05) [5]. OAE is characterized by gastrointestinal symptoms such as severe diarrhea and weight loss, and microscopic findings of enteropathy (villous atrophy) with or without intraepithelial lymphocytic infiltration and a thick band of subepithelial collagen deposition at the duodenal mucosa [4]. However, there are few reports of olmesartan-associated gastroduodenitis (OAGD), which primarily manifests as gastro-duodenal lesions [6, 7]. In addition, no reports have followed the course of endoscopic findings over time, from before the onset of OAGD to its onset, and even after the remission of OAGD. Herein, we report a case of OAGD in a patient presenting with main symptoms of epigastralgia and nausea without diarrhea. Moreover, the detailed course of esophagogastroduodenoscopy (EGD) was examined.

## Case report

A 65-year-old female patient visited our hospital due to symptoms such as epigastric pain, heartburn, and nausea that suddenly developed 6 months back. The patient was treated with vonoprazan at a dose of 20 mg/day for 2 months by a local physician, which resulted in minimal improvement. Her symptoms persisted, and she lost 14 kg. Thus, she was referred to our institution. She did not present with diarrhea. Her previous medical history was remarkable. In particular, she exhibited hypertension, dyslipidemia, and chronic headaches and underwent appendectomy at 18 years of age. She had been treated with vonoprazan 20 mg/day for 6 weeks for a duodenal ulcer, which improved, 2 years prior, and then famotidine at a dose of 10–20 mg/day for occasional heartburn. Subsequently, due to worsening symptoms, the treatment was switched from famotidine to vonoprazan 2 months before her current visit. She had no history of drinking or smoking. Her other medications included olmesartan medoxomil, azelnidipine, simvastatin (each started 7 years ago), as well as mirabegron and imidafenacin (started 6 and 5 years ago, respectively). She had frequently been taking loxoprofen for headaches since she was young. Upon examination, the patient’s body temperature, heart rate, and blood pressure were 36.8 ℃, 85 beats per minute, and 132/69 mmHg, respectively. She had no remarkable chest and abdominal findings. There was no evident superficial lymph node swelling. The blood test results showed low iron levels (mild anemia), slightly decreased serum albumin level, and normal serum gastrin levels. Moreover, she tested negative for serum *Helicobacter pylori* and anti-tissue transglutaminase IgA antibody (Supplementary table).

**Fig. 1:**
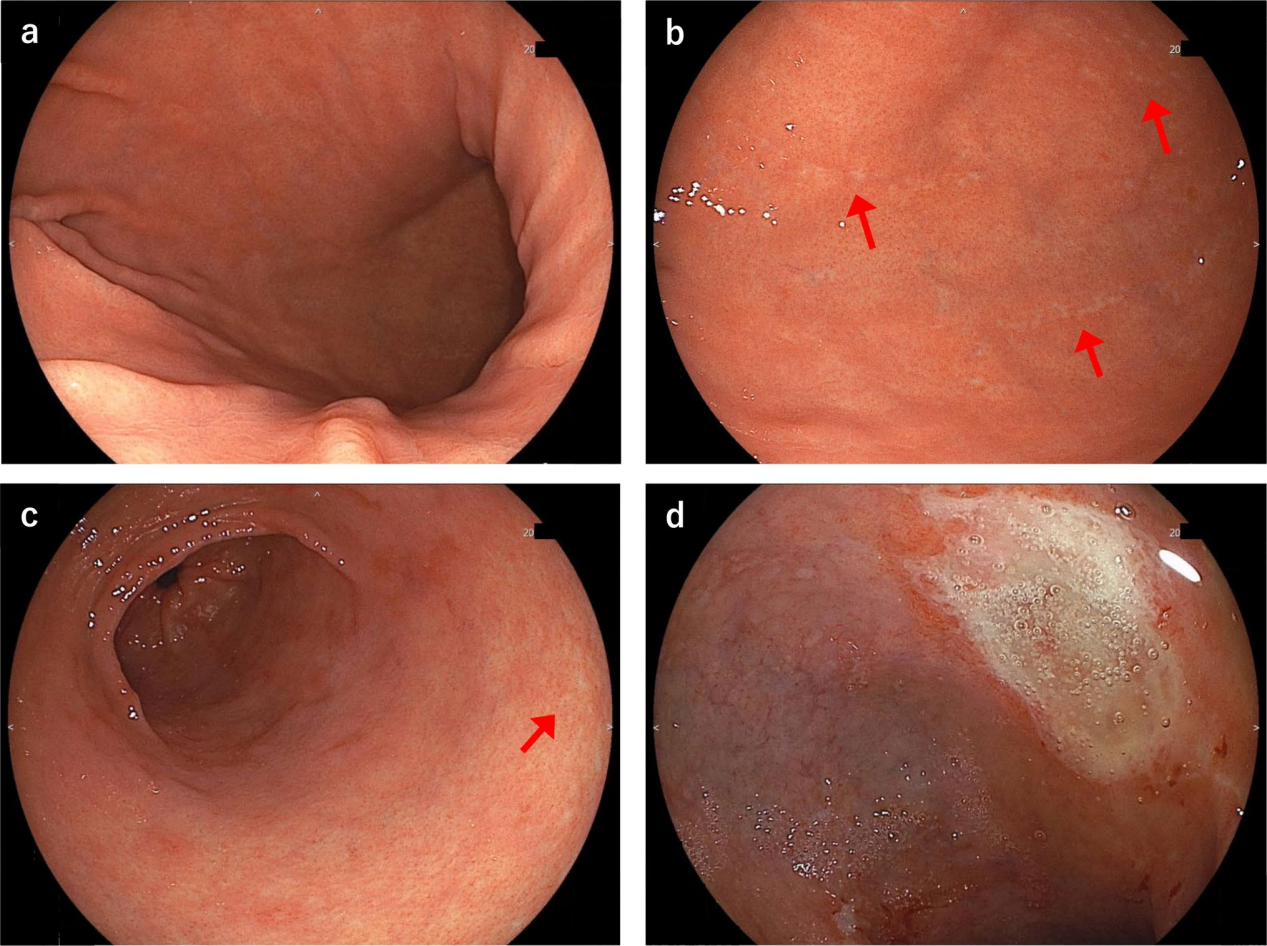
Images of esophagogastroduodenoscopy (EGD) with white light imaging conducted 26 months prior to the patient’s first hospital visit. A normal gastric mucosa was observed in the gastric body (**a**, **b**) and mild atrophic mucosa was partially observed in the greater curvature of the lower gastric body and antrum (arrow) (**b**). Scattered small areas of mild patchy erythema were also observed in the antrum (**c**). A shallow ulcer was observed in the duodenal bulb (**d**)

**Fig. 2:**
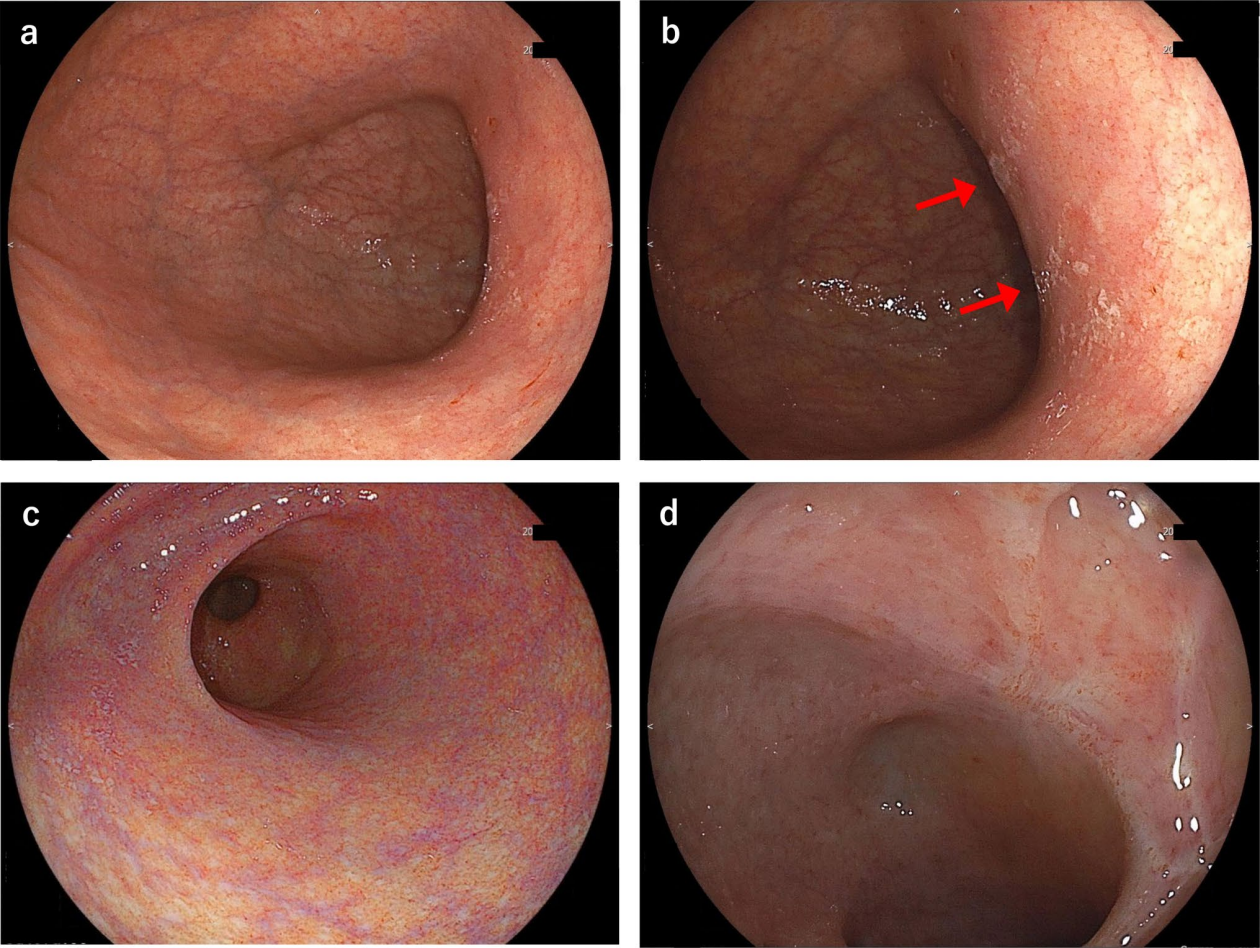
Images of esophagogastroduodenoscopy (EGD) with white light imaging (**a**, **b**, **d**) and linked color imaging (**c**) performed 19 months prior to the patient’s first hospital visit. The area of mucosal atrophy in the stomach extended widely from the gastric body to the antrum (**a**–**c**) and the duodenal bulb (**d**), with a small amount of white adherent material observed in the stomach (**b**, arrow). A healed duodenal ulcer and scar were also observed in the duodenal bulb (**d**)

The patient underwent EGD that was performed by her previous physician 26 months prior to her first visit to our hospital. The results showed minimal mucosal atrophic changes in the stomach, except for small atrophic areas in the greater curvature of the lower gastric body and antrum (Fig. 1a–c). A shallow ulcer was observed in the duodenal bulb (Fig. 1d). Scattered small areas of mild patchy erythema were also observed in the antrum (Fig. 1c). EGD conducted 19 months back revealed a cured duodenal ulcer and scar. However, the area of mucosal atrophy in the stomach extended widely from the gastric body to the antrum and the duodenal bulb (Fig. 2). Further, a small amount of white adherent material was observed in the stomach (Fig. 2b). There was no evident diffuse mucosal reddening, mucosal swelling, or mucosal adhesion in the gastric body.

**Fig. 3:**
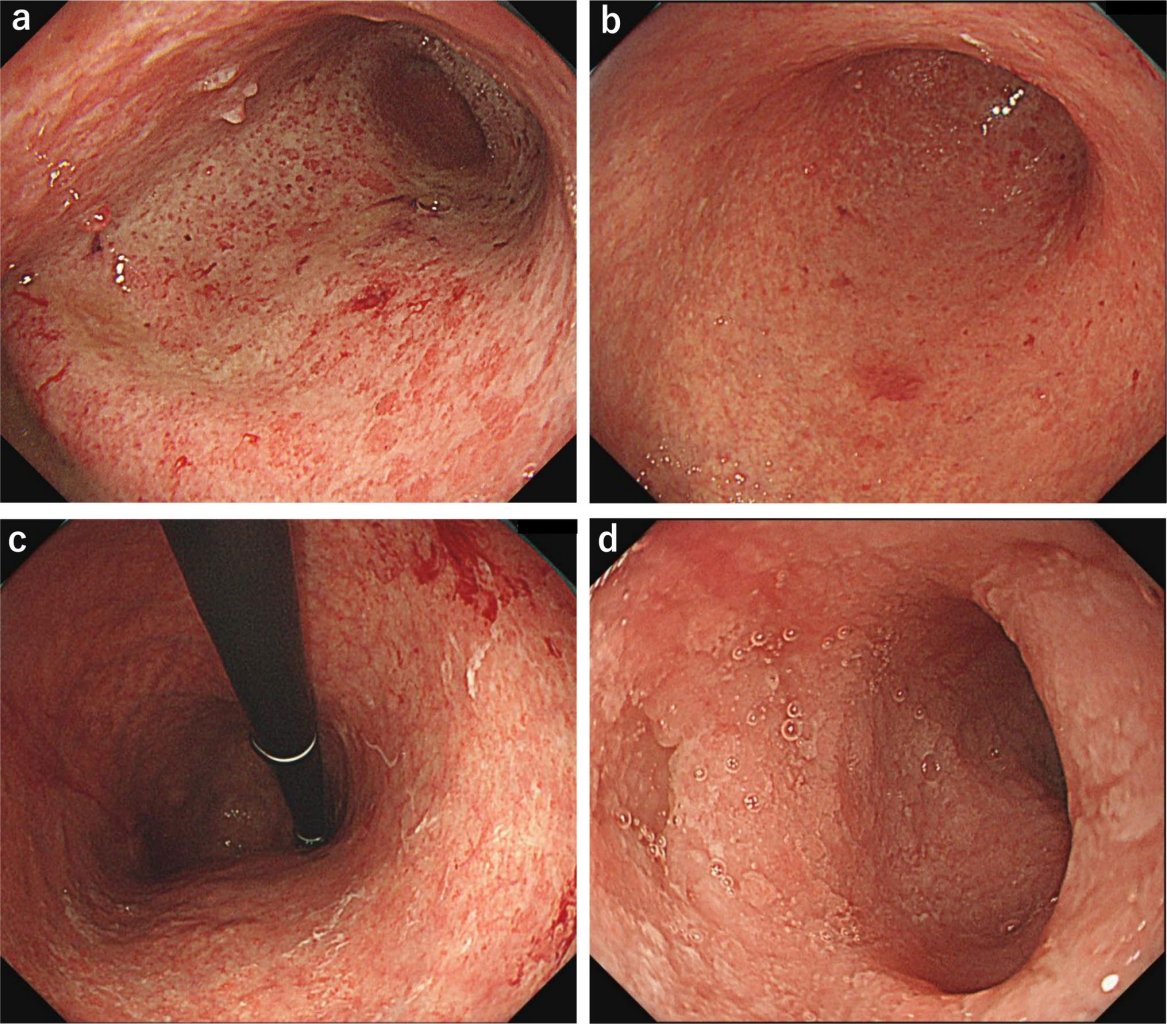
Esophagogastroduodenoscopy (EGD) images obtained at our institution during the patient’s first visit. EGD showed an atrophic gastric mucosa over the whole stomach, and fragile coarse mucosa with hemorrhagic tendency from the middle and lower parts of the gastric body to the antrum, with uniform white purulent adhesions in the same area (**a**–**c**). Diffuse inflammatory changes such as those in the stomach were also observed in the duodenal bulb to the descending part with an ulcer scar (**d**)

EGD performed after her visit to our institution showed an atrophic gastric mucosa over the whole stomach. In addition, fragile mucosa with hemorrhagic tendency from the middle and lower part of the gastric body to the antrum, with uniform white purulent adhesions in the same area, was observed (Fig. 3a–c). The current EGD findings evidently showed an exacerbation of inflammatory findings compared with the previous ones. Diffuse inflammatory changes, such as those in the stomach, were also noted from the duodenal bulb to the descending part with an ulcer scar (Fig. 3d). There were no abnormal findings in the inferior duodenal angulus to the third part (Fig. S1).

**Fig. 4:**
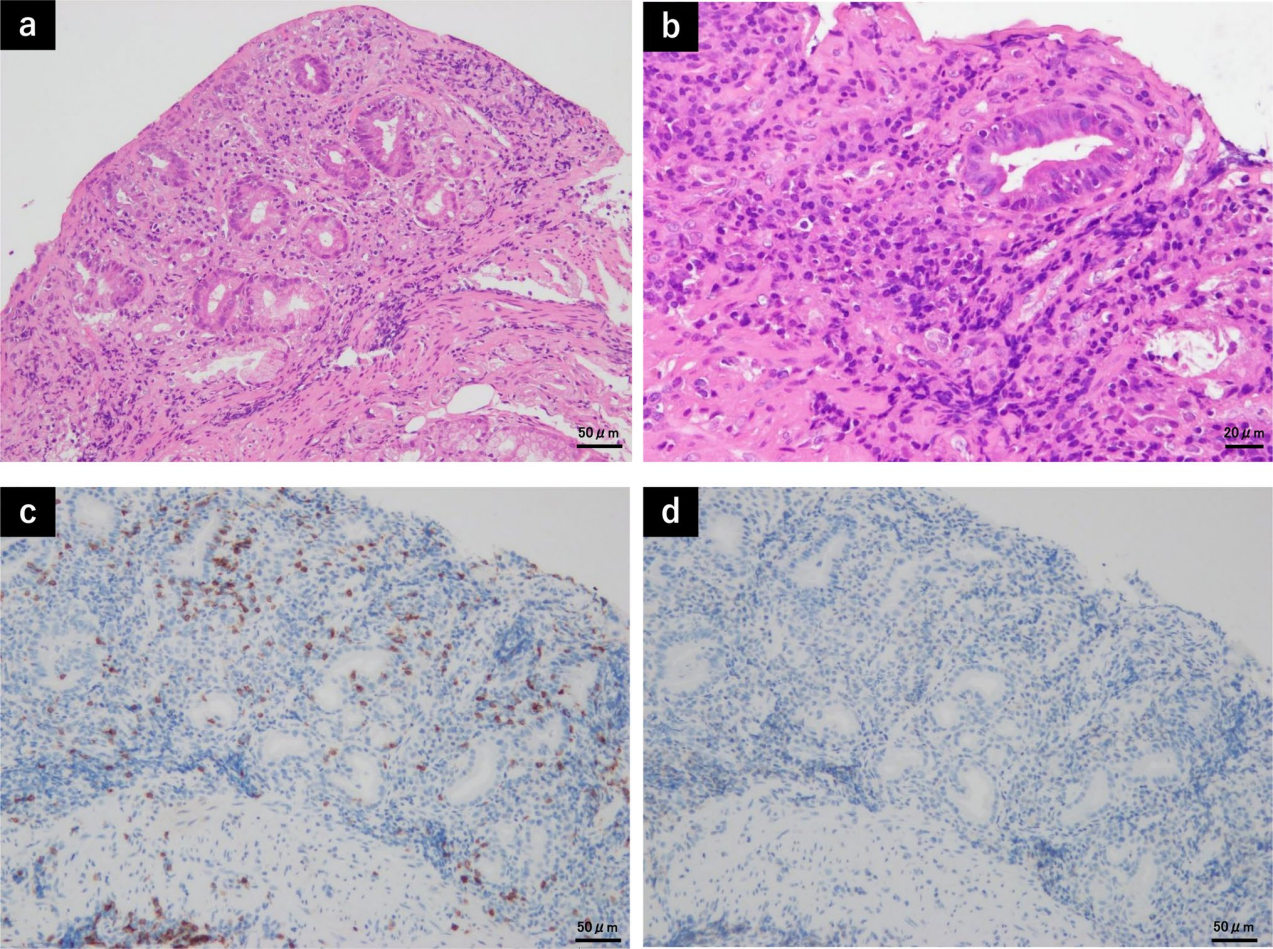
Histological features on mucosal biopsies conducted via esophagogastroduodenoscopy before discontinuing olmesartan treatment. Mucosal atrophic change and inflammatory cell infiltrations in the lamina propria were observed in both the stomach and duodenum (**a**, **b**) Epithelial detachments were also noted in both the stomach and duodenum (**a**, **b**) and the villi structure had almost disappeared and flattened in the duodenum (**a**). In addition, CD8-positive T-cell infiltrations were observed in the gastric epithelium (**c**), without CD4-positive T-cell infiltrations (**d**). **a** Hematoxylin–eosin (H&E) staining (×200), the duodenum. **b** H&E staining (×400), the stomach. **c** CD8 staining (×200), the stomach. **d** CD4 staining (×200), the stomach

Mucosal biopsy performed via EGD at our institution revealed mucosal atrophic changes with adherent exudation in both the stomach and duodenum. In addition, inflammatory cells such as neutrophils, lymphocytes, and plasma cells were found to infiltrate the lamina propria (Fig. 4a, b). Epithelial detachments were observed in both the stomach and duodenum (Fig. 4a, b), and the villi structure in the duodenum had almost disappeared and flattened (Fig. 4a). The collagenous fiber zone just below the epithelium did not thicken, and *H. pylori*-like bacilli or amyloid deposition were not observed. Further, CD8-positive T-cell infiltrations, but not CD4-positive T-cell infiltrations, were observed in the gastric epithelium via immunostaining (Fig. 4c, d). Colonoscopy did not reveal any specific findings from the terminal ileum to the whole colon and rectum. Contrast-enhanced computed tomography scan of the abdomen showed edematous wall thickening in the gastric antrum and several lymph node prominences in the surrounding area. However, there were no evident small intestinal or colonic abnormalities.

**Fig. 5:**
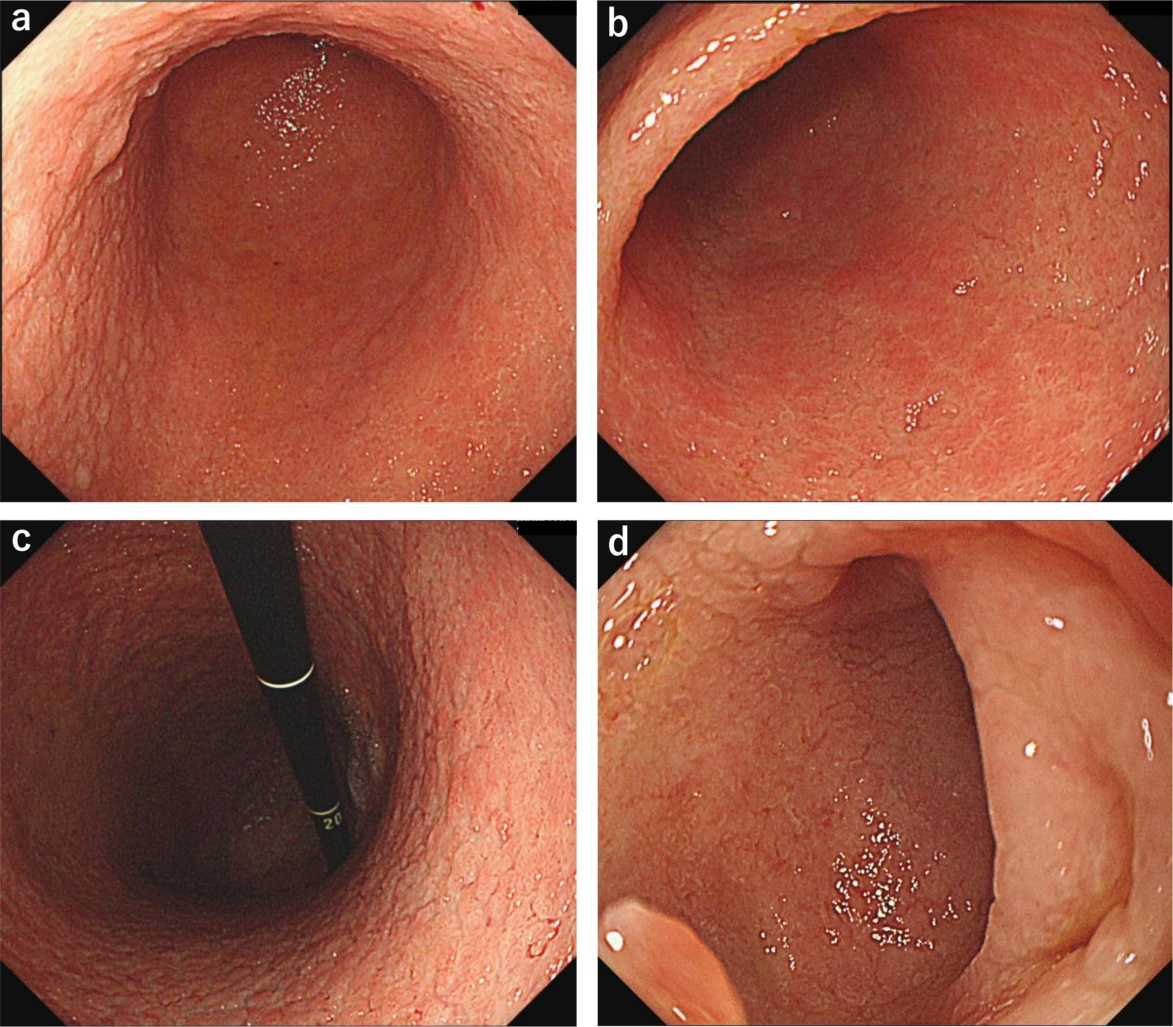
Images of esophagogastroduodenoscopy (EGD) with white light imaging performed 4 months after discontinuing olmesartan treatment. EGD revealed the decrease of purulent mucus and apparent improvement in the inflamed mucosa in the stomach (**a**–**c**). However, diffuse granular change and mild mucosal atrophy persisted from the gastric body (**a**–**c**) to the duodenal bulb (**d**)

**Fig. 6:**
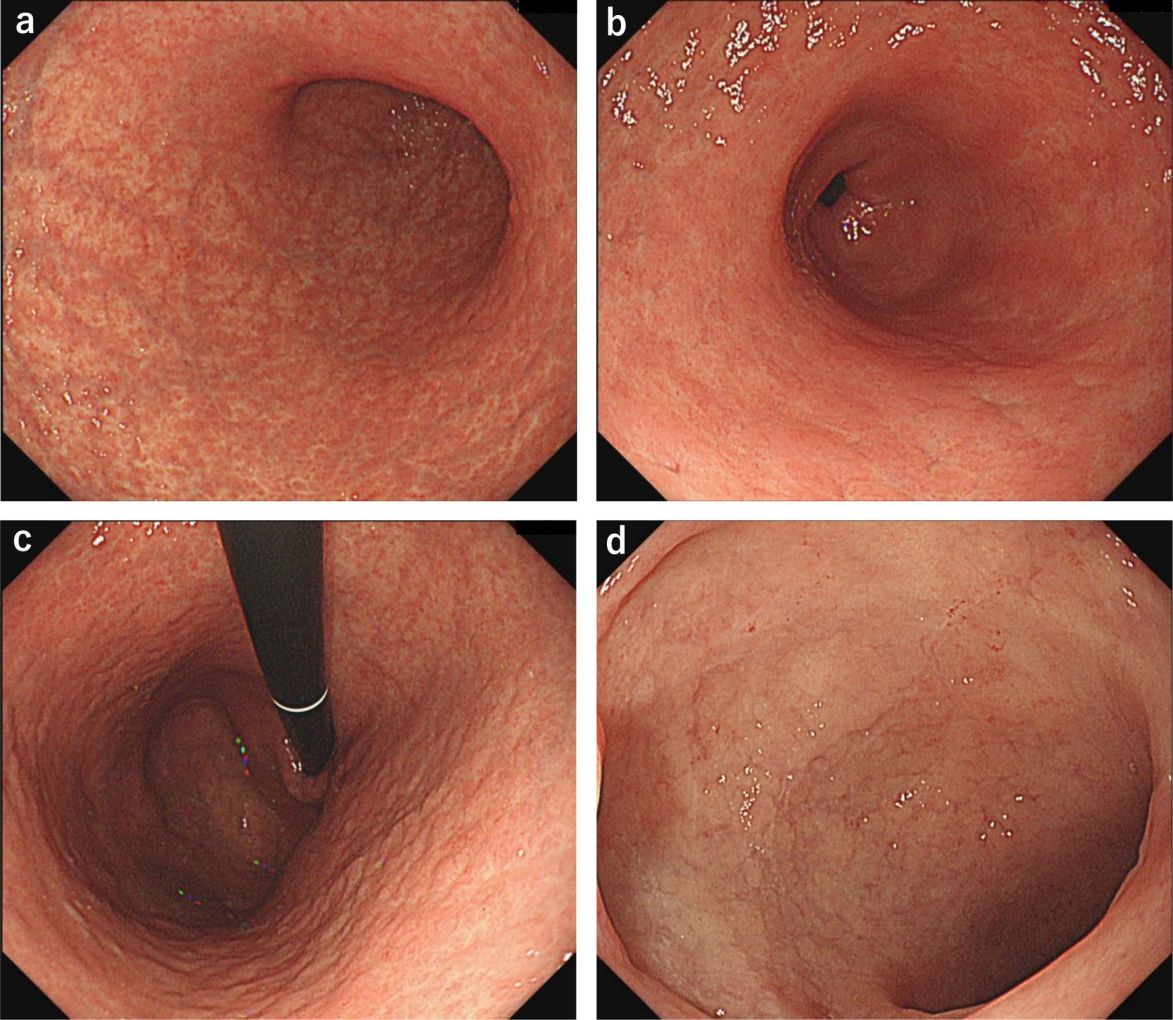
Esophagogastroduodenoscopy (EGD) image with white light imaging performed 16 months after discontinuing olmesartan. EGD revealed minimal mucosal inflammation in the stomach (**a**–**c**) and duodenum (**d**). Meanwhile, mucosal atrophy and roughness were observed in the gastric body (**a**–**c**)

**Fig. 7:**
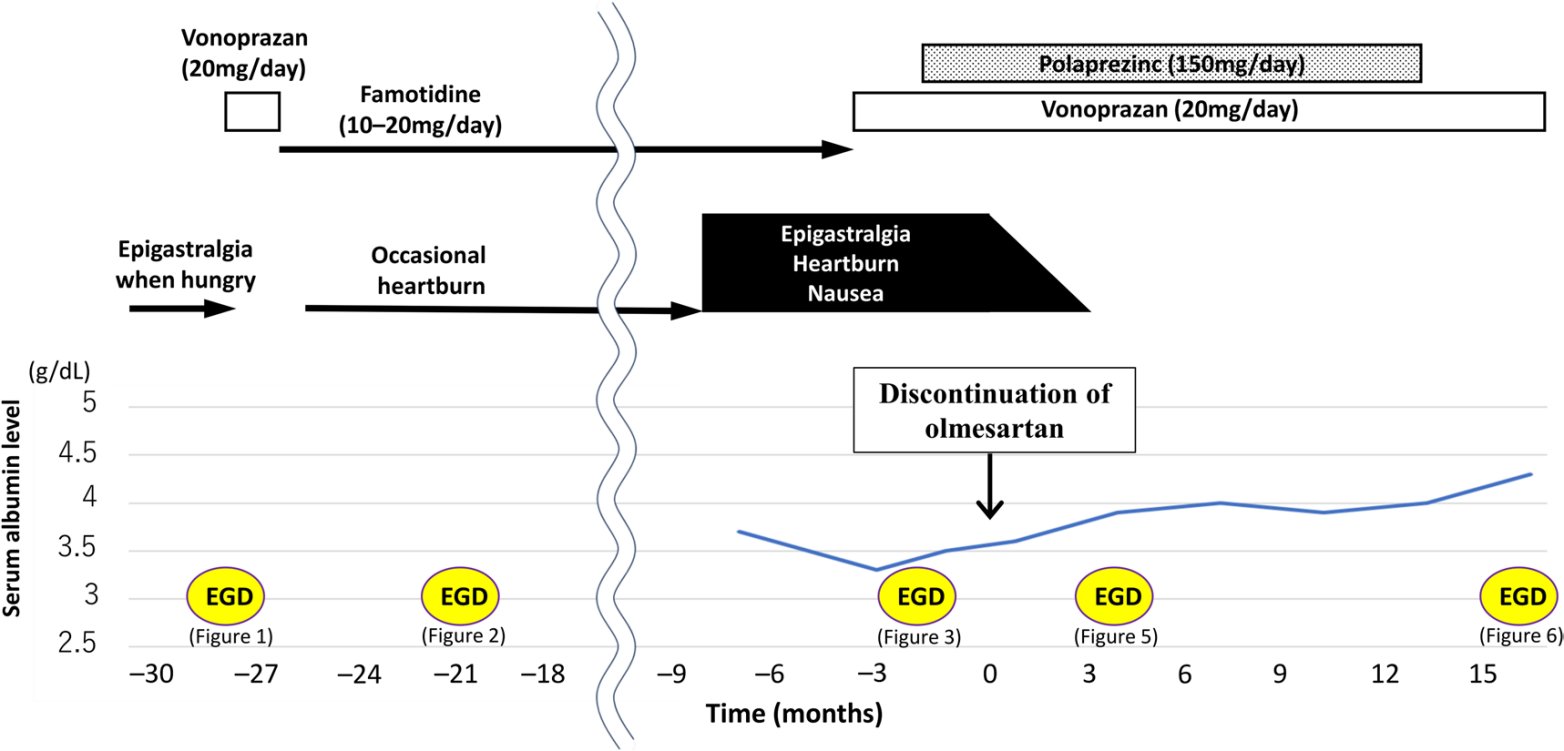
The progression over time, from before the onset of OAGD to after OAGD remission. “0 month” indicates the day olmesartan medoxomil was discontinued. Initially, the patient was treated for a duodenal ulcer with vonoprazan, which improved the symptoms. However, she experienced occasional heartburn thereafter. At 6 months prior to her visit to our hospital, she had been experiencing worsening epigastralgia, heartburn, and nausea. She was then referred to our institution for gastroduodenitis of unknown cause, which did not improve with vonoprazan. Polaprezinc was added, but no improvement was observed. The patient’s symptoms improved after the discontinuation of olmesartan medoxomil, and esophagogastroduodenoscopy showed improvement of gastroduodenitis

The patient’s symptoms did not improve despite discontinuing treatment with non-steroidal anti-inflammatory drugs (loxoprofen) and starting management with polaprezinc in addition to vonoprazan. Gastroduodenitis, characterized by gastric mucosal atrophy and diffuse inflammation extending throughout the stomach, developed within 2 years and could have been drug induced. Given the gradual increase in case reports of enteritis caused by olmesartan in recent years [1–3], discontinuation of olmesartan medoxomil, which the patient had been taking for 7 years, was recommended. The patient then stopped taking olmesartan medoxomil, as well as simvastatin, mirabegron, imidafenacin, and azelnidipine, under the guidance of a local physician. At 1 month after treatment discontinuation, during which the patient came to our hospital, her symptoms and appetite had improved. After another 3 months, the patient’s condition further improved, and she did not exhibit weight loss. EGD performed 4 months after the discontinuation of these drugs revealed decreased purulent mucus and evident improvement in mucosal inflammation in the stomach. However, mold diffuse granular change and mucosal atrophy persisted from the gastric body to the duodenal bulb (Fig. 5). Mucosal biopsy obtained at that time showed severe inflammatory cell infiltrations (mainly plasma cells and lymphocytes) at the lamina propria in the gastric body and duodenum, and mild infiltration of neutrophils and eosinophils in the duodenum. Whereas, mucosal atrophy persisted (Fig. S2a, b). In addition, some endocrine cell micronests (ECMs) were also observed in the gastric body (Fig. S2c). Thereafter, the patient’s condition further improved, and she was eventually cured. She continued receiving vonoprazan (20 mg/day) and polaprezinc (150 mg/day, continued for 13 months after discontinuation of olmesartan) and began treatment with telmisartan and hydrochlorothiazide for hypertension 8 months after discontinuing olmesartan. The patient’s symptoms did not exacerbate, and EGD performed at 16 months after the discontinuation of olmesartan revealed endoscopic findings of minimal mucosal inflammation at the stomach and duodenum (Fig. 6). Moreover, mucosal atrophy and roughness were observed at the gastric body (Fig. 6a–c). Meanwhile, mucosal biopsy showed residual inflammatory cell infiltrations in the mucosal epithelium without ECM (Fig. S3). Following this, treatment with vonoprazan was discontinued, and the patient’s symptoms did not relapse. Figure 7 shows the progression of the patient’s condition. Figure S4 depicts the changes in the gastric mucosa over time, from before the onset of gastroduodenitis, through its onset, and after the discontinuation of olmesartan.

## Discussion

The incidence of sprue-like enteritis caused by olmesartan has been increasing since it was first reported in 2012 [4]. OAE is characterized by diarrhea, nausea and vomiting, and weight loss after initiating olmesartan treatment (median: 19.2 months). The occurrence of diarrhea is often unexplained, and mucosal biopsy from the duodenum occasionally shows villous atrophy and intraepithelial lymphocytic infiltration, and a thick band of subepithelial collagen deposition (collagenous sprues) [4]. It is hypothesized that the pathogenesis of this disease is similar to that of autoimmune enteritis, with immunomodulatory abnormalities playing a role [8]. Moreover, epithelial destruction attributed to increased cytotoxic T lymphocytes (CTLs) is assumed, as mucosal biopsies show an increased number of CD8-positive cells and IL-15 receptors. Further, there may be abnormalities in tight junctions [9, 10].

In a recent systematic review [2] of ARB-related enteritis, diarrhea was reported as a frequent symptom of enteritis in 177 (96.7%) of 183 patients and weight loss in 151 (83.9%) of 180 patients. Meanwhile, of the six cases in this systematic review that showed no symptoms of diarrhea, one case was reported for the first time as OAGD without diarrhea in 2018 [6], and the remaining five cases were only briefly described in large case series [11] and were not reported in detail. Herein, we present in detail a rare case of OAGD without diarrhea. Moreover, to the best of our knowledge, it is the first report on OAGD in which endoscopic findings were observed in detail over time, from before the onset of OAGD to its onset, and even after the remission of OAGD (Fig. S4).

In this case, mild and partial atrophic mucosa was observed in the stomach with duodenal ulcer on EGD performed 2 years before the patient’s first visit. Then, her upper gastrointestinal symptoms worsened, and EGD revealed the development of gastric mucosal atrophy within a short period of time and worsening of gastroduodenitis. Mucosal biopsies showed mucosal atrophy and epithelial detachment in both the stomach and duodenum, with inflammatory cell infiltration in the lamina propria. Moreover, the villous structure had also nearly disappeared in the duodenum. The histologic findings of the stomach and duodenum described above were similar to the previously reported findings of the duodenum in patients with OAE [4]. We suspected that olmesartan caused symptoms worsening because the patient’s symptoms did not improve with medications, and she had been taking olmesartan for a long time.

We consider *H. pylori* gastritis to be deniable for the following three reasons: the patient tested negative for serum *H. pylori* antibodies (< 3 mg/dL); an EGD performed 2 years ago showed mild partial atrophic mucosa, but there were no findings characteristic of *H. pylori* gastritis, such as diffuse redness or mucosal swelling [12]; and all gastric histologic examinations performed during the course of the series showed no evidence of *H. pylori*. In addition, the *H. pylori* stool antigen test was negative. The duodenal ulcer previously observed could have been caused by the frequent use of loxoprofen, a non-steroidal anti-inflammatory drug, as she had used this drug frequently for headaches and experienced epigastralgia when hungry for several months. The patient underwent EGD and was then diagnosed with a duodenal ulcer that resolved immediately with the administration of vonoprazan. However, it cannot be ruled out that the heartburn, which occurred occasionally after the duodenal ulcer had healed, might have been related to OAGD. In addition, gastric mucosal biopsies performed 4 months after discontinuing olmesartan showed some ECMs, indicating the presence of autoimmune gastritis (AIG). However, AIG was also ruled out. This is because ECMs can be observed on gastric biopsy under treatment with proton pump inhibitor or vonoprazan [13]. Further, there was no endoscopic finding of corpus predominantly atrophic gastritis indicating AIG [14], and the patient’s serum gastrin level was normal, which is high in AIG [14]. Subsequent biopsy of the gastric mucosa did not reveal ECM. We also searched the literature for reports on azelnidipine, simvastatin, mirabegron, and imidafenacin as potential causes of drug-induced gastroenteritis, as these drugs had been prescribed by a local physician, but we were unable to find any reports of side effects consistent with the present case for any of these drugs. Since there have been several reports of OAE in the past, olmesartan was the most suspected drug as the cause. Importantly, various symptoms and endoscopic findings in the stomach and duodenum have significantly improved since the discontinuation of olmesartan. Moreover, histologic atrophy of the duodenal epithelium has also mildly improved since treatment discontinuation, which indicates that olmesartan is the most likely cause of gastroduodenitis in this case. Incidentally, telmisartan was started coincidentally for hypertension after the improvement of symptoms in this case. However, the patient’s symptoms have not relapsed to date. In a recent systematic review [2], not only olmesartan (89.6%) but also valsartan (3.6%) and telmisartan (2.2%) were considered causative agents of ARB-related enteritis. Therefore, when starting treatment with other ARBs, it is important to be aware of the potential risk of symptom relapse.

Tanner et al. performed an analysis of EGD in 15 patients treated with olmesartan who presented with abnormalities on gastric mucosal biopsy [11]. This study described the endoscopic findings of the stomach in 13 patients. Results showed the presence of erythema (*n* = 4), mucosal nodularity (*n* = 4), friability (*n* = 2), erosion/ulceration (*n* = 2), polyps (*n* = 2), and atrophy (*n* = 3). In addition, in a case report of OAGD mainly characterized by upper gastrointestinal symptoms in 2018 [6], diffuse erosive gastritis involving the whole stomach and partial duodenitis were observed on EGD, and duodenal biopsy showed epithelial flattening. These findings improved significantly after 3 months of discontinuing olmesartan treatment. Moreover, the gastric mucosa of the patient normalized 6 months after treatment discontinuation. Based on the abovementioned report, it is challenging to determine any endoscopic findings specific for OAGD. However, a case of OAGD in 2018 [6] showed endoscopic evidence of diffuse gastroduodenitis, which clearly improved after discontinuing olmesartan treatment, which is consistent with the current case. In this case, diffuse mucosal atrophy appeared in the stomach within a short period of time, which is a characteristic finding. Considering that duodenal mucosal biopsy in OAE showed a high rate of villous atrophy [4], this finding may indicate gastric mucosal damage caused by olmesartan. In addition, in the current case, the duodenum showed mucosal inflammation extending to the descending part. However, the mucosa of the transverse part on the anorectal side from the inferior duodenal angle was normal within the visible range (Fig. S1). Colonoscopy showed no abnormality in the terminal ileum, colon, and rectum, indicating that the patient’s disease was mainly in the upper gastrointestinal tract.

In terms of histologic findings, in a previous study on 15 gastric mucosal biopsies conducted by Tanner et al. [11], 13 (87%) of 15 patients exhibited active chronic gastritis, of whom 7 (54%) presented with gland atrophy, 4 (31%) with intestinal metaplasia, 7 (54%) with intraepithelial lymphocytosis, and 3 (23%) with subepithelial collagen thickening. In particular, 11 (85%) of 13 patients had surface epithelial injury, a characteristic histologic finding of olmesartan-associated gastritis. Eight patients underwent additional mucosal biopsies after discontinuing olmesartan treatment, with a range of 2–92 months after the initial biopsies. In five of these patients, the histologic results normalized. Another report [15] performed gastric mucosal biopsies on 11 patients with already diagnosed ARB-related enteritis. Results showed that eight patients presented with abnormal histological findings in the stomach, four with metastatic changes, six with neutrophilic activity (active chronic gastritis pattern), and four with a lymphocytic gastritis pattern. These reports show that the incidence rate of mucosal damage in the gastric mucosa of patients with ARB-related enteritis, similar to that in the duodenum, is relatively high. Further, in most cases, mucosal damage can improve immediately after discontinuing olmesartan treatment.

In our case, mucosal biopsies of the stomach and duodenum before discontinuing olmesartan treatment showed villous atrophy, infiltration of inflammatory cells, and epithelial detachment, which are common findings in OAE, as described above. Then, the patient’s symptoms and endoscopic findings immediately resolved after treatment discontinuation. However, mucosal biopsies performed 16 months after discontinuing olmesartan treatment showed residual mild inflammatory cell infiltrations and epithelial atrophy of the stomach and duodenum, which are characteristic findings in our case. In addition, in our case, mucosal biopsy of the stomach showed predominant infiltration of CD8-positive T cells. As previously mentioned, the expression of CD8-positive T cells and IL-15 receptors in the duodenal tissues of patients with OAE significantly increased before the discontinuation of olmesartan treatment [9]. By contrast, in celiac disease, the expression of IL-15 activates cytotoxic T cells and damage epithelial cells [16]. The mechanisms related to celiac disease have been considered in OAE [9], and it is also of interest to note the possibility of a similar mechanism in OAGD.

Herein, we present an extremely rare case of OAGD in a patient with primary symptoms such as epigastralgia and nausea and endoscopic gastro-duodenal findings that changed significantly within a short period of time. Previous reports did not show the characteristic endoscopic findings of olmesartan-related gastritis [11, 17]. However, the atrophic gastric mucosal changes that developed extensively within a short period of time in this case were extremely characteristic and could be one of the findings causing the suspicion of olmesartan-related gastric mucosal changes. Nevertheless, in future, further analysis should be performed, and similar cases must be assessed.

## Supplementary Information

Below is the link to the electronic supplementary material.

Supplementary file1 (DOC 172 KB)

Fig. S1Esophagogastroduodenoscopy image of the inferior duodenal angle to the third part performed at our hospital during the patient’s first visit. There were no abnormal findings in the inferior duodenal angle to the third part (TIF 13612 KB)

Fig. S2Histological features of mucosal biopsies performed 4 months after discontinuing olmesartan. Severe inflammatory cell infiltrations at the lamina propria of the gastric body and duodenum were observed. However, atrophic mucosa persisted (a, b). Some endocrine cell micronests (ECMs) (arrow) were also observed in the gastric body (c). a: Hematoxylin and eosin (H&E) staining (× 200), the stomach. b: H&E staining (× 100), the duodenum. c: H&E staining (× 400), the stomach (TIF 26851 KB)

Fig. S3Histological features of mucosal biopsies performed 16 months after discontinuing olmesartan. Residual inflammatory cell infiltrations within the mucosal epithelium were observed in both the stomach and duodenum (a, b). a: Hematoxylin–eosin (H&E) staining (× 200), the stomach. b: H&E staining (× 200), the duodenum (TIF 19228 KB)

Fig. S4Endoscopic findings of the gastric body and antrum over time, from before the onset of OAGD to after the discontinuation of olmesartan. There was minimal mucosal atrophic change in the stomach, except for slight partial atrophic mucosa in the lower gastric body and antrum 26 months prior to her first visit. However, the gastric mucosal atrophic change progressed widely within a short period of time. At the onset of OAGD, diffuse mucosal atrophic change and fragile, rough mucosa with hemorrhagic tendencies were observed from the gastric body to the antrum. After the discontinuation of olmesartan, the inflammatory findings of the gastric mucosa improved with time, first within 4 months and then at 16 months after treatment discontinuation. However, the diffuse atrophic mucosa in the gastric body and antrum remained (TIF 26185 KB)
