# Supplementary material for: Olmesartan-associated gastroduodenitis that was detected on endoscopic follow-up
Source: Clin J Gastroenterol. 2025 May 4;18(4):563–72. doi: 10.1007/s12328-025-02137-8 (PMC12310766; doi:10.1007/s12328-025-02137-8)
Supplement: Supplementary file 1 — Supplementary file1 (DOC 172 KB) [file 12328_2025_2137_MOESM1_ESM.doc]

| Table S1. Laboratory findings during the first hospital visit | | | |  | |  |  |  |  |  |  |
| --- | --- | --- | --- | --- | --- | --- | --- | --- | --- | --- | --- |
|  |  |  |  | |  |  |  |  |  |  |  |
| WBC count | 3,300 | /µl |  | | Tp level | 6.2 | g/dL |  | Fe level | 40 | µg/dL |
| Neut | 57.3 | % |  | | Glu level | 108 | mg/dL |  | TIBC level | 364 | µg/dL |
| Lym | 35.4 | % |  | | ALP level | 229 | U/L |  | Vit. B12 level | 99 | pg/mL |
| Mono | 1.7 | % |  | | γ-GT level | 17 | U/L |  | Folic acid level | 7.35 | ng/mL |
| Eo | 0.6 | % |  | | T-bil level | 0.5 | mg/dL |  | Ferritin level | 11.6 | ng/mL |
| Bas | 0.1 | % |  | | Alb level | 3.5 | g/dL |  | TSH (ECLIA) level | 1.4 | µIL/mL |
| RBC count | 414 | × 10^4^/µl |  | | ALT level | 6 | U/L |  | F-T3 (ECLIA) level | 2.51 | pg/mL |
| Hb level | 11.3 | g/dL |  | | AST level | 6 | U/L |  | F-T4 (ECLIA) level | 1.56 | ng/mL |
| Ht level | 34.5 | % |  | | LDH level | 168 | U/L |  | IGRA | (-) |  |
| Plt count | 31.7 | × 10^4^/µl |  | | Cr level | 0.64 | mg/dL |  | Serum *HP* antibody level | <3 | U/mL |
|  |  |  |  | | BUN level | 19.1 | mg/dL |  | ANA (FA) | 40 | 倍 |
|  |  |  |  | | Amy level | 62 | U/L |  |  | (speckled type) | |
|  |  |  |  | | CRP level | 0.1 | mg/dL |  |  |  |  |
|  |  |  |  | | Na level | 1434 | mEq/L |  | Serum gastrin level | 130 | pg/mL |
|  |  |  |  | | K level | 4.3 | mEq/L |  | sIL-2R level | 584 | U/mL |
|  |  |  |  | | Cl level | 111 | mEq/L |  | tTG antibody level | (-) |  |
|  |  |  |  | |  |  |  |  |  |  |  |

WBC: white blood cell, Neut: neutrophil, Lym: lymphocyte, Mono: monocyte, Eo: eosinophil, Bas: basophil, RBC: red blood cell, Hb: hemoglobin, Ht: hematocrit, Plt: platelet, TP: total protein, Glu: glucose, ALP: alkaline phosphatase, γ-GT: gamma-glutamyl transpeptidase, T-bil: total bilirubin, Alb: albumin, ALT: alanine aminotransferase, AST: aspartate aminotransferase, LDH: lactate dehydrogenase, Cr: creatinine, BUN: blood urea nitrogen, Amy: amylase, CRP: C-reactive protein, Na: sodium, K: potassium, Cl: chlorine, Fe: serum iron, TIBC: total iron-binding capacity, Vit.: vitamin, TSH: thyroid-stimulating hormone, ECLIA: electrochemiluminescence immunoassay, F-T3: free triiodothyronine, F-T4: free thyroxine, IGRA: interferon gamma release assay, *HP*: *Helicobacter pylori*, ANA: antinuclear antibody, FA: fluorescent antibody, sIL-2R: soluble interleukin 2 receptor, tTG: anti-tissue transglutaminase IgA
