# Supplementary figures and images for: Olmesartan-associated gastroduodenitis that was detected on endoscopic follow-up
Source: Clin J Gastroenterol. 2025 May 4;18(4):563–72. doi: 10.1007/s12328-025-02137-8 (PMC12310766; doi:10.1007/s12328-025-02137-8)

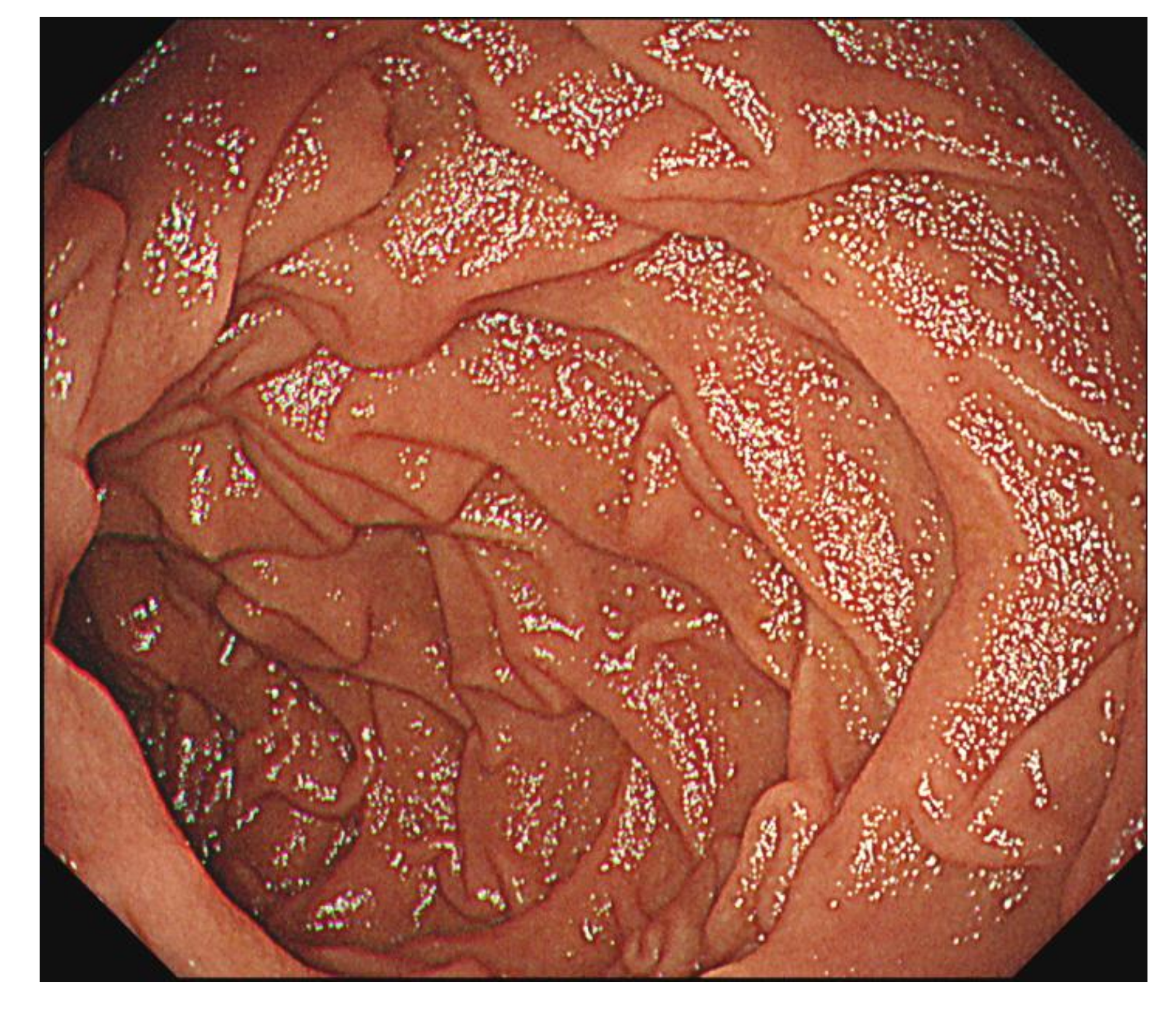

Supplement: Supplementary file 2 — Fig. S1Esophagogastroduodenoscopy image of the inferior duodenal angle to the third part performed at our hospital during the patient’s first visit. There were no abnormal findings in the inferior duodenal angle to the third part (TIF 13612 KB) [file 12328_2025_2137_MOESM2_ESM.tif]

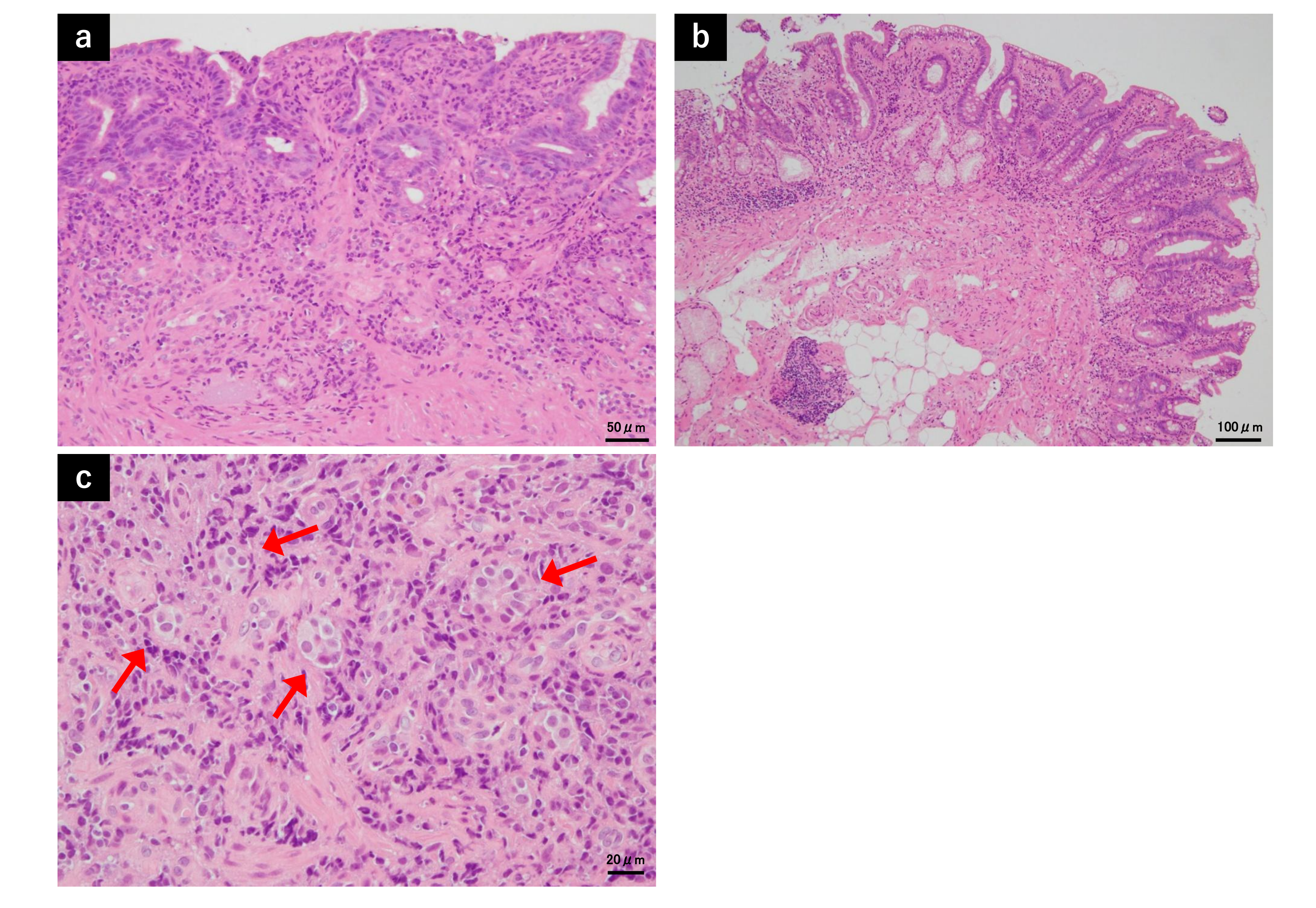

Supplement: Supplementary file 3 — Fig. S2Histological features of mucosal biopsies performed 4 months after discontinuing olmesartan. Severe inflammatory cell infiltrations at the lamina propria of the gastric body and duodenum were observed. However, atrophic mucosa persisted (a, b). Some endocrine cell micronests (ECMs) (arrow) were also observed in the gastric body (c). a: Hematoxylin and eosin (H&E) staining (× 200), the stomach. b: H&E staining (× 100), the duodenum. c: H&E staining (× 400), the stomach (TIF 26851 KB) [file 12328_2025_2137_MOESM3_ESM.tif]

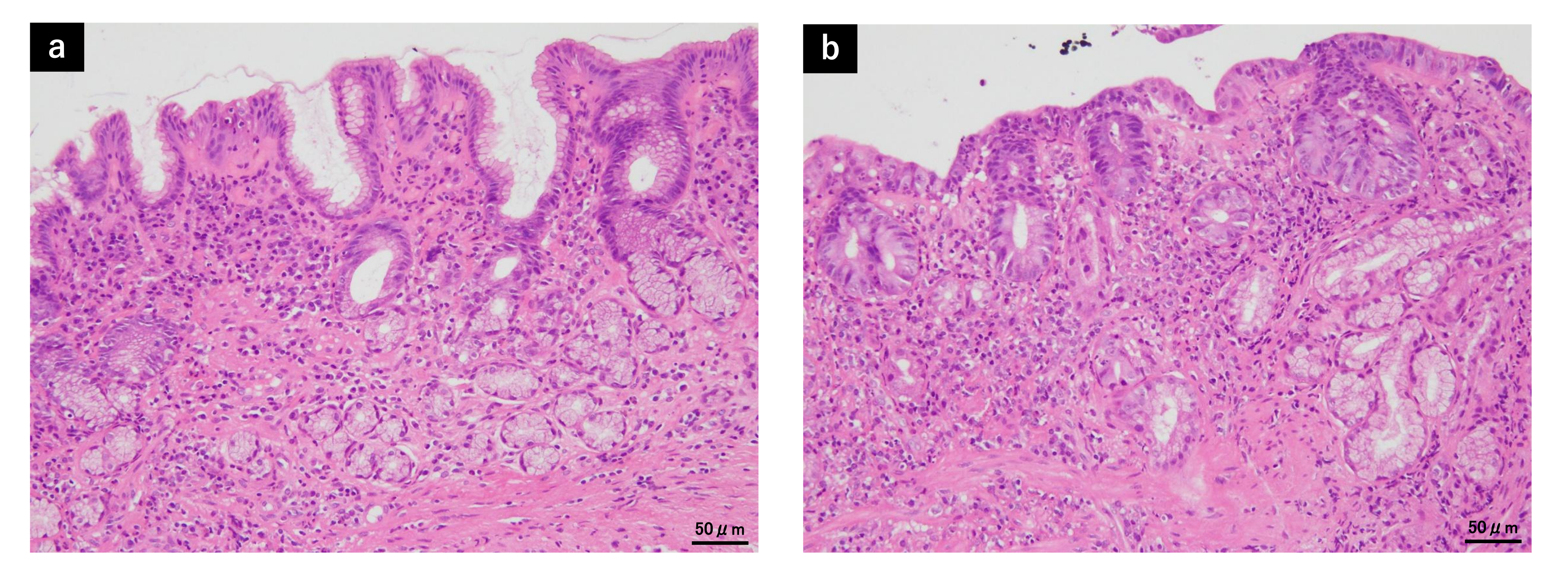

Supplement: Supplementary file 4 — Fig. S3Histological features of mucosal biopsies performed 16 months after discontinuing olmesartan. Residual inflammatory cell infiltrations within the mucosal epithelium were observed in both the stomach and duodenum (a, b). a: Hematoxylin–eosin (H&E) staining (× 200), the stomach. b: H&E staining (× 200), the duodenum (TIF 19228 KB) [file 12328_2025_2137_MOESM4_ESM.tif]

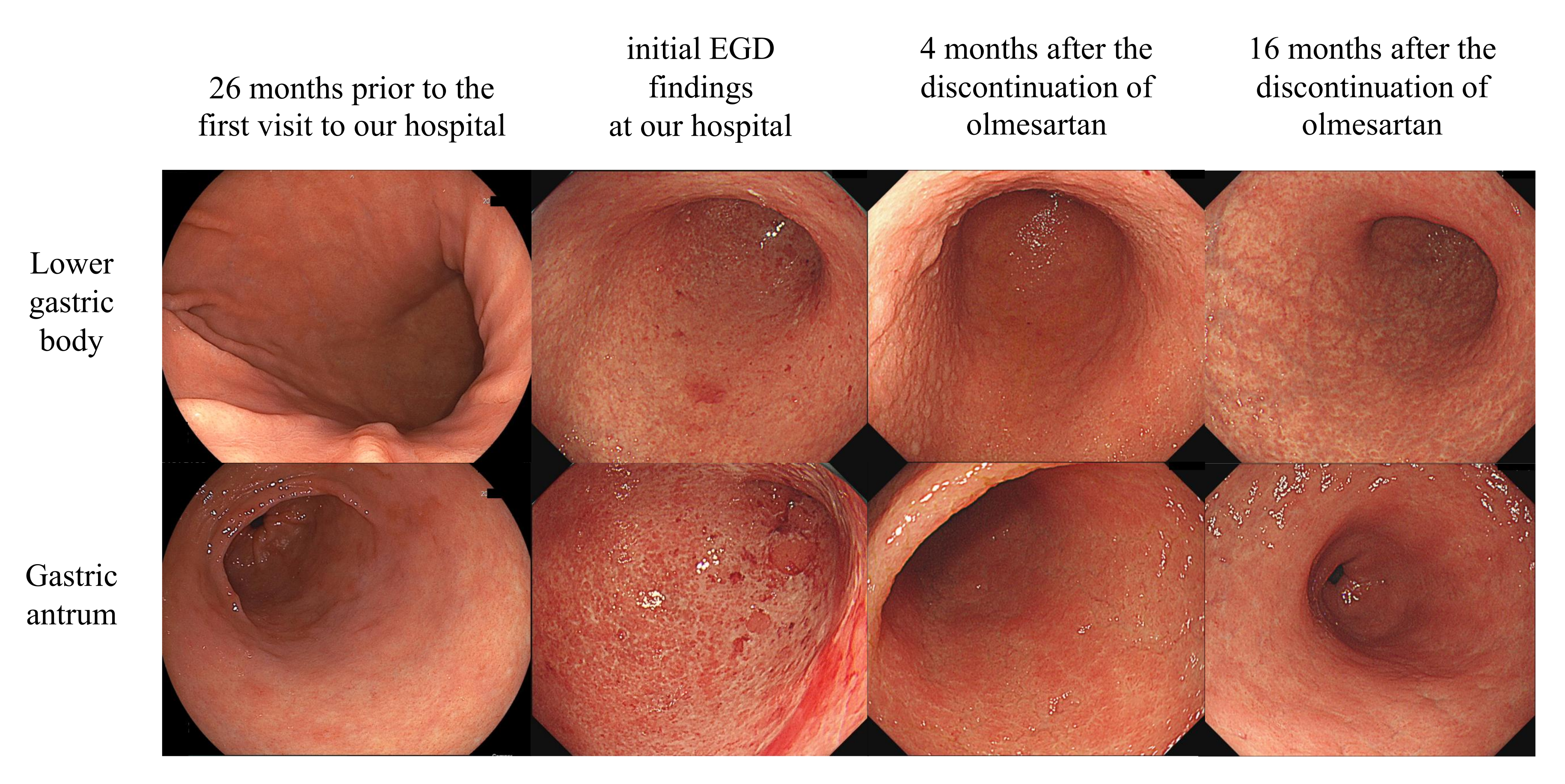

Supplement: Supplementary file 5 — Fig. S4Endoscopic findings of the gastric body and antrum over time, from before the onset of OAGD to after the discontinuation of olmesartan. There was minimal mucosal atrophic change in the stomach, except for slight partial atrophic mucosa in the lower gastric body and antrum 26 months prior to her first visit. However, the gastric mucosal atrophic change progressed widely within a short period of time. At the onset of OAGD, diffuse mucosal atrophic change and fragile, rough mucosa with hemorrhagic tendencies were observed from the gastric body to the antrum. After the discontinuation of olmesartan, the inflammatory findings of the gastric mucosa improved with time, first within 4 months and then at 16 months after treatment discontinuation. However, the diffuse atrophic mucosa in the gastric body and antrum remained (TIF 26185 KB) [file 12328_2025_2137_MOESM5_ESM.tif]
